# Correction: Impact of hospitalization on nutritional status in persons aged 65 years and over (NUTRIFRAG Study): Protocol for a prospective observational study

**DOI:** 10.1371/journal.pone.0327678

**Published:** 2025-07-02

**Authors:** Cristina Carretero-Randez, María Isabel Orts-Cortés, Margarita Rodríguez-Pérez, Víctor Manuel Gonzalez-Chordá, Eva María Trescastro-López, Joan Blanco-Blanco, Jordi Martínez-Soldevila, Aránzazu Ruiz-Heras-Hera, Pedro Raúl Castellano-Santana, Amando Marquez-Sixto, Manuela Domingo-Pozo, Antonia Inmaculada Zomeño-Ros, Jesica Montero-Marco, Marta Charlo-Bernardos, Joaquín Moncho, Ángel Luís Abad-González, María Trinidad Castillo-García, Ana Belén Sánchez-García, Ana María De Pascual y Medina, Rosa Ana Clement-Santamaría, Ascensión Franco-Bernal, Rafaela Camacho-Bejarano

The word “Nutrifag” is misspelled in the article title. The correct title is: Impact of hospitalization on nutritional status in persons aged 65 years and over (NUTRIFAG Study): Protocol for a prospective observational study. The correct citation is: Carretero-Randez C, Orts-Cortés MI, Rodríguez-Pérez M, Gonzalez-Chordá VM, Trescastro-López EM, Blanco-Blanco J, et al. (2023) Impact of hospitalization on nutritional status in persons aged 65 years and over (NUTRIFAG Study): Protocol for a prospective observational study. PLoS ONE 18 (11): e0288348. https://doi.org/10.1371/journal.pone.0288348.
